# Book Reviews

**Published:** 1825-01

**Authors:** 


### CRITICAL ANALYSIS
OF
### ENGLISH AND FOREIGN LITERATURE
RELATIVE TO THE VARIOUS BRANCHES OF
### Medical Science.

Quæ laudanda forent, et quæ culpanda, vicissim

Illa, prius, cretâ; mox hæc, carbone, *notamus*.—PERSIUS.

### DIVISION I.

### ENGLISH.

ART. I.—*Lectures on Digestion and Diet.* By CHARLES TURNER THACKRAH, Member of the Royal College of Surgeons of London, of the Société de Médecine Pratique de Paris.—London, 1824; pp. 156.

THE author of these Lectures is already favourably known to the profession by his "Inquiry into the Nature and Properties of the Blood, as existent in Health and Disease;" and the circumstance of the work before us coming from the same pen would, of itself, dispose us to take early notice of it, even if the subject of which it treats had less claim to our attention. In perusing this volume, however, it must be borne in mind that it is not intended exclusively for the professional, but likewise for the general reader; a circumstance attended with at least one advantage, that no attempt is made to display extraordinary erudition,—or, to give the author's own words, "ignorance is not clothed in a learned phraseology, nor supported by a parade of authorities." If, therefore, we meet with nothing remarkable for its novelty, so neither are we led to expect it; and if any one should open the volume with this hope, he will assuredly be disappointed. But we confess that it appears to us quite as useful to give a distinct and connected detail of all that is most important upon any subject, as to impose upon the attention by bringing forward doctrines more remarkable for their ingenuity than their practical utility; a fault which too much characterises the writings of the present day.

The dependence of the animal frame upon a due performance of those functions which are destined to extract chyle fit for its nourishment, from our infinitely varied kinds of food, is too obvious to require any illustration; and with regard to the interest of the subject, that which is so intimately connected with the daily pleasure of one portion of mankind, and the daily penance of another, can scarcely be a matter of indifference to any,—certainly not to any who know what it is to pass a portion of each day " dans les horreurs de la digestion."

It is only of late years that the changes which the food undergoes in its passage through the body, have received the degree of attention they merit, or been converted to purposes of a practical nature. For much of what we know of digestion, we are indebted to the philosophic mind of Hunter, which could never rest content with mere speculative doctrines, however plausible, nor with experiments performed without method or rational object, however difficult their execution might be; but whose experimental researches were made the basis of legitimate, and hence of useful, inductions. Considerable practical advantage has certainly resulted from the attention which has of late years been directed to the digestive functions, in the treatment of disease; and of this the chief merit cannot be refused to Mr. Abernethy, even by those who are of opinion (and we confess ourselves to be of the number,) that his doctrines have frequently been carried to an unwarrantable, if not to an absurd extent. We allude, in particular, to the system of generalisation, which would place the cure of all diseases under the sun in the administration of blue-pill and rhubarb; a vice which, as is often the case, we believe, is to be attributed more to the disciples than the master.

But, to come to the work of Mr. Thackrah, we find it divided into three parts, whereof the first contains an account of Digestion, preceded by some general physiological remarks; the second relates to Diet; the third to the Diseases of the Alimentary Canal; and of these we shall speak in order, having first afforded the author an opportunity of developing his own views, as follows:—" My object in these Lectures, is to give a general, but not incorrect, view of physiology,—to diffuse useful information in a popular form. I have, therefore, endeavoured to make the language as plain, and the illustrations as intelligible as possible. The Lectures will not be found a mere compilation. Most of the facts and doctrines have been matter of reflection, and not a few of direct experiment. The deeper researches of physiology are precluded by the nature and limits of the course. Nor will disputed doctrines be mentioned, unless they seem of particular importance. I shall endeavour to explain, not what we doubt or deny, but what we know or believe. The examination of functions has naturally led me to notices of disorders. Hence I have briefly adverted to the principal diseases, their causes, and sometimes also the means of prevention. To these notices I had added a sketch of the medical treatment; but this, except in a few instances, has since been erased." (P. 7.)

After a general description of the apparatus of the gullet and stomach, we find some important observations connected with the gastric juice. It has generally been supposed, since the experiments of FORDYCE and others, that meat which had become tainted was capable of being sweetened in the stomach. This is contrary to the observations of Mr. Thackrah.

"1824. Jan.—I found in the stomach of a cat, putrid meat, which had been eaten long before,—probably seven hours. I requested my pupil, Mr. Horton, to give to a cat, which had been without food since the preceding day, a piece of flesh, as putrid as could be procured at this season of the year. An hour afterwards, on examining the contents of the stomach, we found the fœtor increased rather than diminished. To a dog was given a piece of meat, decidedly putrid: after three-quarters of an hour, he was pithed, and the stomach opened. The fœtor of the meat was not diminished in the slightest degree.

"The discordance in the observations of Fordyce and myself, may arise from a circumstance which I remarked in another experiment. When the stomach is opened immediately after death, the rising halitus diffuses so strong an animal odour, as wholly to cover that of the food. Hence, in a hasty notice, no fœtor is perceived, even when the flesh, on a closer examination, or on its removal from the stomach, is found extremely offensive." (P. 14.)

The author believes that the gastric juice is capable of acting upon the stomach, under particular circumstances, as stated by Hunter; and mentions an instance of his having found perforation in the stomach of a child, who had died of hydrocephalus. We suspect, however, that this must rather have been one of the cases alluded to in Mr. NORTH's paper in our last Number.

Returning to the process of digestion, we find the following—rather fanciful description of the office of the pylorus:

"After the elaboration of the food, the longitudinal fibres of the muscular coat bring the lower aperture of the stomach into a line favouring the transmission of the gastric product. But, before it can enter the intestines, it must necessarily pass the pylorus. The office of this door-keeper is not a sinecure. He must examine the qualifications of every applicant, and allow those only who are in a suitable state to pass his portal. Accordingly, the muscular ring contracting, drives back all undigested matter, and compels it to perform again the round of the stomach. It appears, however, that the pylorus, like other officers, may, by repeated solicitation, be induced to transgress his orders; for clasp-knives (as in the instance which I lately mentioned), half-pence, and I believe also pence and crowns, have been sent through the aperture. It is related that Vaillant, when pursued by corsairs, swallowed twenty valuable gold medals, which at length passed the canal; and that he even sold one of them by anticipation, before it had made its appearance. Several substances also, difficult of solution, but harmless either from their nature or size, are permitted to pass: sometimes, indeed, are early thrown into the intestines, in order, as it would seem, that the stomach might employ its energies on food more soluble or nutritious." (P. 19, 20.)

In attributing so extensive a guardianship to the pylorus as a repellent of undigested matter, we presume the author alludes particularly to those substances which are too large to admit of a ready transit; for the irritation we so frequently witness from undigested food passing into the intestines, (one of the most common causes of diarrhœa,) clearly proves that the pylorus is not always a very vigilant officer.

The subject of *gastric digestion* being completed, we come to that which takes place in the *intestines.* In this part we observe nothing with which our readers are not familiar. The author thinks that chyle is absorbed in the large as well as the small intestines, having himself seen it in the horse, and quoting the authority of Dr. Schwenke, who found the mesocolon "interspersed with numerous well-filled lacteals" in a soldier, who was killed by a ball, which entered the thorax, and lodged in such a manner as to compress the thoracic duct, thus preventing the ascent of the chyle. We confess that we are surprised that the author, in treating of chylification, should have omitted to notice the very important fact recently mentioned by Mr. Brodie, with regard to the influence of the bile over this part of digestion: we presume it has escaped him.

Speaking of the rapidity with which certain substances manifest their presence in the secretions, the author adverts to the conjecture of some physiologists, that a direct passage exists, by which they find their way into the circulating mass. But, however striking the phenomenon may be, the recent experiments of some of the French physiologists, particularly M. Fodera, appear to us to prove, in a satisfactory manner, that, even when the transit is most rapid, it is effected without any other mechanism than that of the general absorbent system.

As connected with the subject of digestion, we find some inquiries into the causes and phenomena of hunger and thirst, which, if not new, are at least interesting.

"The deductions, which these considerations present, shall be compressed into as few words as possible. Hunger, in common with all sensations, is immediately dependent on the nerves. But the question is, what gives the primary impression to the nerves of the stomach? Various hypotheses have been offered, but the present state of our knowledge scarcely warrants the adoption of any. The most probable opinion is that which ascribes it to the gastric juice. This fluid, of powerful character, is constantly secreted. During digestion, it is mixed with the food; but, after the cessation of this process, the juice is accumulated on the sensible surface of the stomach, which it peculiarly irritates.

"This theory may, perhaps, account for most of the phenomena.

The cold to the skin increases hunger, by diminishing the secretions on the surface, and thus augmenting those of the internal organs,—consequently those of the stomach. Stimuli, as bitters and acids, can improve the appetite, only by augmenting or amending the secretion of gastric juice. The compression of the stomach suspends hunger, as great and continued pressure reduces the action of the nerves. Narcotics act on the stomach as they do on the nervous system in general. Emotions, also, by drawing off the nervous energy, preclude the sensations of the stomach, like those of the other organs. Food, immediately on its commixture with the gastric juice, abolishes the appetite; for the inner surface of the stomach is no longer irritated by the accumulated fluid. Whatever, in short, increases the gastric juice, increases also the appetite; whatever reduces the quantity or quality of this secretion, reduces also the appetite. Hence savages, so often destitute of food, remove the cravings of hunger by lime, magnesia, or other earths, and the Kamtschatkadale by swallowing saw-dust." (P. 34, 35.)

"Whatever reduces the secretions in general, draws off the thinner parts of the blood, or determines this fluid, and its essential concomitant, the nervous energy, to other organs than the throat and mouth, produces or augments the sensation of thirst. Hence the distress from the abstraction of liquids. Hence the thirst from copious and ardent perspiration; the thirst of nurses; and that also of dropsies dependent on increased action of the exhalants. Hence, too, the common thirst of inflammation. Violent fits of passion are often succeeded by urgent thirst; for the nervous energy apportioned to the secretions, is expended on the emotion and the actions to which it gives rise. A similar agency, I conceive, renders thirst the most severe part of the punishment of torture. The cry of the wretch on the rack is 'Drink, drink!'

"Thirst is reduced most speedily by bleeding. This, I conceive, acts by subduing the inflammatory excitement in the throat. The bath and affusion of water mitigate thirst; probably by the sympathy of the alimentary canal, and consequently of the throat, with the surface of the body. It is relieved not only by taking liquids, and thus directly moistening the heated and constricted parts, but even by rolling in the mouth any smooth substance, as a pebble or tamarind-stone. This, of course, is a mechanical stimulus to the secretion of saliva and mucus. Acids quickly reduce thirst: they too, perhaps, act as stimuli. Water alone affords by no means the most speedy relief. In hot climates, it is rapidly carried off by perspiration, and, according to a remark before made, the increase of secretion on the skin necessarily diminishes that of the throat. Hence the addition of wine or spirit, by preventing the transpiration of the water, tends to quench thirst much more completely.

"'Thirst,' says Magendie, 'is an internal sensation, an instinctive feeling; it belongs essentially to the organisation, and admits of no explanation.' In the present state of our knowledge, no theory, perhaps, can be firmly established; but the facts to which I have adverted lead to the opinion, that the state of the nervous and vascular system, termed thirst, arises from *a want of secretion in the mouth and throat.*

"Both hunger and thirst appear to be of local origin. They indicate, indeed, the state of the system at large; but the sensations themselves arise in the stomach and throat. They correspond with the wants of the whole economy; but they do not seem to depend on these wants." (P. 36—38.)

Some of the best cases on record of *continued abstinence* are related, particularly the well-known narrative of Captain Bligh and his companions, and another from an early volume of the Philosophical Transactions; but the one possessing most interest, is the history of the German merchant, given by Hufeland in his Journal about two years ago. After a cursory review of some of the peculiar circumstances attending digestion, the author terminates his first Lecture with a paragraph, setting forth the general influence of the stomach, in which our physical, and even moral, dependence upon that viscus are placed in rather a mortifying light.

The second Lecture commences with some observations on the various kinds of *aliment.* The digestive apparatus of animals which feed on vegetables, is compared with that of carnivorous animals; which ends in the conclusion most rational people have agreed in,—that man, having the teeth of the simiæ, the stomach of the lion, and the bowels of the ourang-outang, combines the devouring propensities of all these, and is omnivorous. This leads us to some remarks upon particular kinds of diet exclusively.

" Notwithstanding these facts, vegetable food has been recommended as the only proper sustenance of man. We certainly find a vegetable regimen highly useful in certain cases of disease, or disposition to disease. In epilepsy, and in some affections of the head, and in most diseases of the heart, I have employed it with marked utility, and in some disorders of the digestive organs with minor advantage. In gouty habits, it has sometimes been highly useful. There are, moreover, peculiar constitutions, which, though apparently healthy, will not bear a diet of flesh. In some individuals, the stomach even nauseates the idea.

" Two cases have recently fallen under my notice, in which the individuals, without professional advice, adopted a diet of vegetables.—Mr. W. tells me, that, suffering long under bilious disorders, and obtaining little relief from medical treatment, he tried a strict regimen of vegetables and water. His health and spirits, he assures me, have since been greatly improved; and he is, consequently, a warm advocate of the herbivorous system. But, within the last two years, he has judiciously added to his dinner a moderate proportion of flesh.—A gentleman from B., who had been under my care for a chronic disease, was induced, soon after his recovery, to try the vegetable system. After its use for some months, he informed me that it had removed an oppression from the head, which, though slight, had been before almost constant; that his general comfort was increased, and his strength by no means reduced. But lately I learn that he, as well as Mr. W., has added meat to his dinner of herbs. In neither of these cases do I doubt the advantage at first received, but I conceive that it was not imputed to the true cause.

"These observations, with many of a similar kind, might be usefully compared with the statements of Dr. Lambe and Mr. Newton. The full examination of the subject would prove, I believe, that, though a strict vegetable regimen, in certain peculiarities of constitution, and in some states of disease, is highly advisable,—almost essential to life and health,—it is by no means the only sustenance which suits the healthy organs of man.

"The system has been recommended for the mind, as well as the body. A strict diet of vegetables is said to improve the intellect. Sir I. Newton, it is well known, when writing his Treatise on Optics, abstained from flesh. Dr. Lambe states that his rigid regimen has increased considerably his intellectual powers. In Watts' work on Diabetes is detailed the case of a student, the subject of severe depletory treatment, whose intellect was raised in the inverse proportion to the reduction of his body. From my own observation, also, I am convinced that by low diet the intellect is often relieved and invigorated." (P. 61—63.)

But we have the counterpart of this:—

"On the other hand, I have seen it improved by an opposite system. A young gentleman, who has tried various diets, assures me that his mind is most capable of exertion when his body is most plentifully nourished. He now takes flesh twice, and milk once a-day. Dr. Stark, on taking beef after a low diet, had 'a keenness for study.' On inquiry, I learn that at Cambridge low diet is unknown. It is said that the senior wrangier of a late year took a large quantity of flesh. Dryden and Fuseli, to exalt their imaginations, ate raw meat." (P. 63.)

It appears probable that the digestive process, in different individuals, eliminates very different quantities of nourishment, even from the same food; so that a kind and quantity of diet, which is sufficient for one, may be by no means so for another. The vigour of mind appears connected, to a considerable extent, with the arterial circulation in the brain; and while one, having the vessels overloaded by animal diet, is relieved by vegetable food, so another may require the stronger aliment for the formation of a sufficient quantity of blood. According to our author, even the temper may be influenced: thus, he has known a gentleman, who lived for some months on vegetables, have his temper rendered much less irritable; while another, of a different disposition, became more irascible on reducing his diet. Arbuthnot says, "I know more than one instance of irascible passions being much subdued by a vegetable diet." It was in allusion to this idea of modifying the disposition by diet, that Galen desired the teachers of philosophy to send persons of bad character to him, in order to be cured.

We entirely agree with our author, that mixed food is the kind best fitted for most people in a state of health; and that considerable deviations from this, unless in certain states of disease, are apt to be injurious. We deprecate, especially, the system of living, or rather of starving, upon vegetables; as if God Almighty would have given us the same sort of teeth and stomach, to live on vegetables alone, which he has bestowed on the dog, and other animals which eat flesh. We insist the more upon this point, from having recently seen some ghost-like figures among our cabbage-eating friends, whose rueful visages give the lie to their doctrine—that the excitement of animal food is unnatural, and hence prejudicial. The recent occurrences at the Millbank Penitentiary have given a useful lesson upon this subject. They remind us of the man who reduced the allowance of his horse to a single straw, and then wondered that he died.

"The elements of chyle are evidently more abundant in animal than in vegetable food. Marcet found the chyle of carnivorous creatures to be whiter, to contain more solid matter, and to yield more albumen, than that of the herbivorous. This observation we should anticipate, from considering the nature of animal food, and the approximation of its principles to those of the product of digestion. Gelatine, albumen, and fibrine, the chief constituents of flesh, approach nearer to the nature of chyle, than starch, sugar, and gluten, the chief constituents of vegetables. Azote is the principal element in the animal fabric. Flesh, therefore, yields it abundantly; while in vegetable substances it is scanty, and in many, as urged particularly by Magendie, wholly absent. When, however, this gas is not provided in the food, it may, perhaps, be derived from the atmosphere, by the lungs or skin. In articles of animal food, there is less variety than in those of vegetable. Though greatly differing in their digestibility, almost all yield abundant chyle. Muscle, on the whole, is the most valuable.

"If, then, we find good chyle to be produced both from vegetable and animal food, the question is, which affords it in the proportion and circumstances best suited to the individual. This, in fact, though overlooked by the advocates of a particular regimen, is the great dietetic inquiry." (P. 67, 68.)

There are various judicious, but not new, remarks upon the different degrees of solubility of different substances; and we shall, therefore, pass on to the following philippic against cheese:—

"Cheese varies less in its degree of digestibility, than in its nutritious and stimulating qualities. It is with difficulty changed by the solvent fluids; and hence, in large quantities, it is very pernicious. Lately, I was called to a lady, of an apoplectic disposition, who was well and cheerful in the evening,—ate a hearty supper of toasted cheese,—was seized soon after with a fit,—called for assistance,—became violently convulsed, and spoke no more. Instances like this are by no means unfrequent. A person goes to bed after a hearty meal, and is found in the morning dead or paralytic. The hearer is surprised and affected at the tale,—moralises on the uncertainty of life and health; but rarely inquires into the cause,—rarely inquires what course of life gave the disposition to disease, and what oppression of the stomach induced the catastrophe." (P. 78.)

Now, although we by no means advocate the propriety of eating large quantities of toasted cheese, either at supper or any other meal, yet we do think that an equal quantity of any other food would probably have given rise to the same symptoms. It is the habit of eating plentiful suppers generally, rather than indulgence in any particular article, that seems to give rise to apoplexy so frequently under these circumstances.

Moderate diet is strictly enjoined by our author, and no one will deny the justice of the recommendation; but, unfortunately, men's opinions differ not a little touching this moderation. That most men eat more than is absolutely necessary, we verily believe; but, at the same time, we are of opinion that the system of starvation is as bad, if not worse. We never meet with such obstinate cases of disease as among some of the lower orders, who are compelled to practise moderation in their diet from necessity, not choice. We think, too, that the little work of Cornaro, which the author quotes, would by no means answer as a guide by which to judge of the quantity of food requisite. This self-satisfied old gentleman was, indeed, an exception from mankind; for he asserts that he found, what Rasselas never could in all his travels—the way to be happy. It is obvious that much of what was attributed by him to the diminution of his diet, is to be attributed to the natural hilarity of his disposition.

"The hermit," says Mr. Thackrah, "derived from his restricted diet not only health, but I conceive also those pleasures of imagination, which could only compensate the want of social intercourse. Pleasant dreams, it is well known, may be obtained by attention to food and habits; and it is probable that the raptures and visions of happy enthusiasts were, in no small degree, dependent on their scanty diet. I recollect no instance of a happy enthusiast being a gormand or an epicure.

"We wish to live, not only as long, but as much and as happily as possible. A man, whose brain is oppressed, scarcely lives as an intellectual being; a man with a nauseant stomach, has not even animal enjoyment. Yet we start at the idea of being confined to a system of temperance, as if it were confinement in a house of correction. We have a vague, but appalling notion, that it abridges the enjoyments of life. But, if this notion be brought to the test of fact and reason, we shall assuredly find that temperance not only lightens and cheers the major part of the day—the great bulk of our existence, but promotes even the pleasures of the table." (P. 92, 93.) no doubt of [illegible]

It has generally been supposed, particularly since the experiment of Professor Harwood on two pointers, that exercise immediately after eating has the effect of impeding very much, if not entirely arresting, the process of digestion. Our author has made some experiments upon this subject, the results of which do not exactly correspond with those alluded to.

"1. To a hungry dog was given three ounces of food, flesh and bread in equal quantities. The animal was kept walking for three hours, and then killed by pithing. The contents of the stomach, though copiously moistened with gastric juice, were two drachms less than the food eaten. We may, therefore, infer (as the gastric juice would add considerably to the weight,) that from one-sixth to one-fourth had been digested. The major part of the aliment was little changed in appearance.—2. Another dog was fed in a similar manner, and suffered to be at rest. At the end of three hours, on examining the contents of the stomach, we found them not more changed than in the preceding experiment, but three drachms less than the aliment.—3. An experiment similar to the first, except that the animal was kept in easy motion for four hours. The contents of the stomach weighed four drachms less than the food eaten. They were universally pervaded by the gastric juice, and the bread was entirely broken down.—4. In the next experiment a large dog, fed in the usual manner, was kept at rest for four hours. The contents of the stomach did not weigh three drachms; digestion, consequently, was almost complete. If this animal had not been much larger and stronger than the others, perhaps the two last experiments would have more nearly approximated. Horrid cruelty

"The universal habit of labourers, in resuming their employ soon after dinner, confirms the inference from experiment. If digestion must wait for the repose of the body, their meals would remain in the stomach for five or six hours, and gastric disorder would ensue. The argument I need not pursue. I would remark, however, that the conclusion applies only to the healthy organs, acting on a moderate quantity of aliment. If either the organs be weak or the food excessive, repose is necessary to digestion. Hence the dyspeptic is advised to sit an hour or two after dinner, with no other occupation than conversation or a light book. Either mental or muscular exertion would draw off that nervous energy which digestion demands. A jolting motion is particularly injurious: it displaces the food, and interrupts the action which should subject successive portions to the gastric juice." (P. 99, 100.)

The inactivity of body and mind which prevails after eating, appears to us a sufficient indication of the general necessity of rest and quietude for a certain period: yet we believe that exercise, under certain circumstances, may be advantageous. We remember to have heard the late celebrated Dr. G., of Edinburgh, assert, that the Professors of one northern college lived much longer than those of another in their vicinity; and he assigned as an explanation, that, "after their great eating don't eat so as to make exercise disagreeable to you and drinking suppers, the Professors of Marischal College tumbled into bed immediately; while the others had to walk a mile and a half before they reached home,—had time to get sober,—and so escaped indigestion and apoplexy."

The author next treats of various kinds of *drink*, and among others of tea,—a beverage which it has been so much the fashion to condemn, that scarcely any medical man ventures to speak favourably of it. Mr. Thackrah says, it is "naturally injurious;" and a little further on asserts, that its effects in most persons are "rather useful than prejudicial." How it should be naturally injurious, we are at a loss to know, and believe, on the contrary, that the latter part of the sentence, though contradictory of the former, is correct: at least, we are sure that, if poisonous, it must be (as Dr. JOHNSON said) a very slow poison indeed.

This Lecture concludes with an eulogy upon the memory of the late Dr. STARK, in which we cannot altogether agree with the author. In the experiments of this unfortunate young man we observe nothing but the self-devotion of an enthusiast; and no results, which could rationally be looked for from his experiments, could justify the means. Science gained nothing by his sacrifice; and the example is one to be shunned, although we pity the unfortunate victim of his own wild and absurd experiments.

The Lecture on the *disorders of the alimentary canal*, opens with a passage which we shall quote, as it places anatomy in its proper point of view; and we know that many students are too apt to fancy their medical education completed as soon as they have obtained a tolerable knowledge of the structure of the body, when in point of fact it is but just beginning: they have learnt the alphabet only, and must next learn to read.

"The prosecution of physiology requires anatomy, human and comparative,—experiments on the functions in health,—and observations on states of disease. This may be deemed an established truth; but the subject is not generally considered in the extent of its relations, nor has it been practically regarded with adequate zeal. Anatomy is the lowest grade of the study, the mere vehicle of science,—absolutely necessary, indeed, but no more forming a professional character than a knowledge of the alphabet constitutes the scholar.

"Observations, and especially experiments on animals, are highly important, when they fairly interrogate nature, and are conducted by a reflecting and accurate mind. But the deductions of comparative physiology are not always adequate to give complete satisfaction on the doctrines of human physiology: they sometimes require collateral observations on man himself. But these, it is apparent, can rarely be obtained by direct experiment; they must be collected in the rounds of general practice. Causes, moreover, are sometimes best investigated by examining them in relation to their effects; and the ordinary functions are sometimes best illustrated in the studies of Pathology. Physiology, then, ought not to be an insulated study, either in the sources of its facts, or the application of its doctrines. It should go hand-in-hand with surgery and medicine, and accompany the practitioner in his daily routine.

"Nor is the subject of less moment in professional education. The student must not rest in an acquaintance with the individual branches. He must learn to trace their relations,—to deduce his therapeutical principles from the doctrines of disease; and these again he must found on the knowledge which the hand of anatomy displays, and the mind of physiology examines, compares, and elaborates." (P. 114, 115.)

This chapter contains many sensible remarks, but not of a nature to be embodied in our analysis,—with one exception. It is a note on the subject of administering purgatives in enteritis, which so much agrees with the opinions which we have elsewhere inculcated, that we shall extract it; and, in doing so, we terminate our quotations.

"Purgatives are very frequently the first remedies administered for inflammation of the bowels. From this practice I have seen the worst effects. In true enteritis it is highly hazardous, and can rarely be useful. The object of purgation is to remove the constipation. But this state is not the cause of the inflammation; it is merely an effect, and by no means a dangerous effect. What is purgation? An increased action of the exhalants, and an increased action of the muscular coat. In enteritis, the action of the exhalants is suspended, and the muscular coat, we believe, in a state of inflammatory excitement. If, then, the exhalants be urged to pour out fluid, when their mouths are closed by disease,—if the muscular coat be urged to drive hard substances through a canal morbidly sensible,—we must anticipate the aggravation of the disease, or even the induction of gangrene." (P. 133.)

The work, upon the whole, contains little that is new, but much that is true; and the only advice we have to give the author is, in another edition, to have it printed in a more rational manner: we allude to the absurd way in which spaces are left between the sentences. —for example.

Art. II.—*Report of the Epidemic Cholera, as it has appeared in the Territories subject to the Presidency of Fort St. George. Drawn up by order of the Government, under the Superintendance of the Medical Board.* By William Scot, Surgeon, and Secretary to the Board.—Madras, 1824; folio, pp. lxxii, 292. *With an Appendix; containing the Sick-Returns of the Army of Fort St. George, from the year* 1815 *to* 1821, *inclusive, exhibited in a series of fifteen Tables; also Diurnal Tables of the Progress of Cholera, in six Parts; Tables of the Movements of the Troops, and Meteorological Tables, in seven Parts, from* 1815 *to* 1821.

We have been favoured, by the kindness of a friend, with the perusal of the above highly interesting and ably written documents; a kindness which we estimate the more, as there are but few copies of the book to be met with in England, and those only in private hands. The work is very voluminous, and complete in all its parts: no point of interest that could be supposed to throw any light upon the formidable, and to us little understood, subject of epidemic cholera, appears to have been neglected; and we conceive a careful perusal of its contents to be the indispensable duty of all those who are about to undertake the practice of the medical profession in India.

It is our intention to present our readers with as extended an account of this Report as our limits will permit; a task which does not, upon a nearer view, appear so formidable as we first feared it would have proved, since the greater part of the volume is occupied with the Reports from the different military and civil medical officers, whose fortune it was to witness this disease; and the paper drawn up by Mr. Scot, and standing at the head of this volume, is an epitome of the whole correspondence, and a very able and impartial account of the disease, as afforded by an attentive review of these documents. It is, in fact, itself a critical account of the whole question, which we cannot do better than follow as our surest guide. The Correspondence, upon which this Report is founded, has a peculiar value, because it is drawn from so many sources, and written under so many different circumstances, both of time and place, as to do away all suspicions of previous agreement or understanding between the parties; so that where we find all, or the greater part, of the medical observers testifying to the same facts or appearances, we become assured of their being founded in truth, and our knowledge of the disease, and its best mode of treatment, become established upon clear and unerring grounds. Annexed to the work is a map of the Peninsula of India, on which the principal places visited by this epidemic in 1818-1819 are marked, with the periods at which it made its appearance in its progress from north to south; commenccing at Nagpoor, in May 1818, and arriving at Trivandrum, in January 1819.

The recent invasion of cholera in India, appears to have excited the most serious and general alarm. It at first seems to have been looked upon as the visitation of some new and unknown plague; therein resembling the sensation created by the attack of the Walcheren fever in the British army, in the year 1809,—a disease which took all our medical men by surprise, but which it was after recollected had been described by Sir J. Pringle, by Dr. Windt, and numerous other authors. Such we shall find to be the case with the cholera of India: but we now hasten to Mr. Scot's Report.

After a page or two of prefatory matter, in which our author explains the circumstances under which the present volume was produced,—circumstances which are as honourable to the governing powers, as creditable to the zeal and ability of the medical department in India,—and something like an apology for the alteration or compression of some of the Medical Reports, Mr. Scot proceeds to say, that, "although no paper is inserted in this Report which is not official or authenticated, nor any thing advanced, unless founded on materials exclusively of that nature, yet it is nevertheless to be remembered, that medical *facts* are too often merely medical opinions: their authenticity, with reference to the views of the narrator, is undoubted; but, liable as even the most deliberate and sagacious observers are to error and misconception, we are bound to receive with due caution and circumspection any *facts*, or *opinions*, which are palpably at variance with general experience." We have inserted this passage as a proof of the spirit of cautious and philosophical inquiry, which pervades the Report itself.

We next come to the circumstance adverted to above. Cholera was at first supposed to be an entirely new disease, and its previous existence, nay, its description by European authorities, appear still to be reluctantly admitted, although proved beyond the possibility of doubt. In the proceedings of this very Medical Board itself (Madras), under the date 29th November, 1787, this disease is most accurately described, in all its formidable and peculiar symptoms, as having prevailed at Arcot in 1770, in the Ambore valley in 1783, at Ganjam in 1781, under the name of Cholera Morbus, or Mordyxim. Passing over some Hindoo authorities, and that of the Dutch physician Bontius, together with some writers contemporary with the invasion of the disease in 1770, we find that its visitations were extended to the Mauritius, in the year 1775. It is noticed also by Curtis, who served in the fleet of Sir E. Hughes, where it is described by the name of Mort de Chien, or Cramp; the first word being, to our minds, an evident corruption of the Indo-Portuguese word

Morydixim, or Mordeshim; since the French language never, to our knowledge, acquired so much ascendancy in India as to have left any traces of its existence. Whoever has taken the pains to read the account as given by Curtis, can entertain no doubt of the disease he describes being the true epidemic cholera. It is needless to accumulate authorities. Drs. GIRDLESTONE, DUFFIN, and DAVIS, respectively give accounts of different invasions of the disease. It is alluded to by Dr. J. JOHNSON; but seems from that period to have sunk almost into oblivion, though it is incidentally mentioned in one or two military Medical Reports, in the year 1814; but, its ravages having been confined to a battalion or two, and appearing only sporadically, it does not seem to have attracted much attention. From the inspection also of the Army Returns, cholera appears to have prevailed more or less, from year to year, among the troops, whilst in certain districts of the country. It has also, at various times, been endemic among the natives, especially at Travancore, where it obtained the name of Neer-comben.

We have said enough, we conceive, to prove that cholera is no new disease in India.

We shall omit the remarks of Mr. Scot upon the different names, both scientific and vulgar, that have been given to the disease by different medical authorities. He objects, we think reasonably enough, both to CULLEN's and Dr. GOOD's nomenclature, and proposes the distinctive term of Cholera Asphyxia, as descriptive of that symptom which may be called essential to the disease. Mr. Scot does not appear to be aware that SAUVAGES has a distinct species of cholera in his nosological arrangement, called Cholera Indica.

Leaving this discussion, however, we proceed to a description of the malady itself; and here we must recollect that difference of season, of local situation, perhaps of food, and many other circumstances that will readily suggest themselves to the medical reader, will fully account for the many shades of difference observable in the character of the reports of the symptoms and mode of attack; whilst there is a remarkable agreement as to two or three never-failing phenomena which constitute the true pathognomic signs of the disease, and are mentioned by all the reporters without exception. The approach of cholera is not indicated by any premonitory symptoms; for a slight nausea or laxity of the bowels, occasionally met with, cannot be so designated, since they are common to so many other complaints. The disease very generally begins in the night, and towards morning the patient vomits the contents of his stomach, and the bowels are at the same time evacuated. This evacuation is peculiar to the disease: the whole canal appears as if at once emptied of its contents, and a sudden feeling of exhaustion is produced, faintness supervenes, the skin becomes cold, there is giddiness and very often deafness; spasmodic twitchings of the muscles of the legs and arms are felt, extending towards the trunk of the body; the pulse from the first is small, weak, and accelerated, and very soon, and especially upon an accession of the spasms, becomes imperceptible in all the external parts, the skin becomes colder and colder, but is usually covered with a clammy moisture; in Europeans the color is livid, the lips blue, and the nails of the same hue; in this state the skin is insensible even to chemical agents, yet *heat* upon the surface is always complained of. The eyes are sunk in the orbits, the features of the face collapse, and the countenance becomes cadaverous. Thirst is an urgent symptom, but the tongue is moist, whitish, and cold; there is usually a distressing sense of burning heat at the epigastrium, the secretions are very much lessened or totally suppressed; the breathing is oppressed and slow—the breath is deficient in heat.

During the progress of the disease, the intestinal canal is variously affected:—after the first evacuations, the matter discharged is watery, most commonly colourless and inodorous; in some cases it is turbid, resembling muddy water; occasionally it is greenish or yellowish: the stools usually called "conjee stools," are produced by numerous mucous flakes floating in the watery part of the evacuation, and the discharge from the stomach does not appear to differ from that of the bowels, excepting in their being mixed with the ingesta. The vomiting and purging are not of long continuance, and, together with the spasms, they generally disappear a considerable time before death. Towards the close of the disease, restlessness, internal anxiety, and distress, come on, and death takes place from ten to twenty hours from the commencement of the attack. During the whole of this period the mental functions are undisturbed even to the last: a favourable issue is denoted by rising of the pulse, a return of heat to the surface, inclination to sleep by a cessation of the spasms, vomiting and purging, the re-appearance of fœcal matter, and a return of the secretions of bile, urine, and saliva. In speaking of the varieties of this epidemic, Mr. Scot premises that these varieties are not so much observed in *individual* cases, as in *local epidemic visitations;* but the most fatal variety is found to be that in which all the above symptoms can scarcely be detected, but a mortal coldness, and arrest of the circulation, come on from the beginning, the patient dying without a struggle. These modifications have been observed unconnected with any local peculiarity of weather, food, shelter, or occupation. In tracing each separate symptom of Cholera, we find that the major part of them may be wanting, and yet the disease be really as clearly of the same nature and as rapidly fatal as when the assemblage of morbid phenomena is present in the succession mentioned above; for instance, it happened that *vomiting* has been entirely absent upon many occasions; nor is this absence always a favourable sign; indeed, it is considered that where it is either not present or soon ceases, so that the stomach retains whatever is poured into it, this condition is most alarming.

It is a subject of doubt and dispute what it is that imparts the yellowish or greenish colour to the matter ejected from the stomach: it has been supposed to be imparted by bile; but this is extremely doubtful, according to our author, though we cannot rely much upon some experiments detailed in a note, and we must leave the matter in the state we find it; but we may remark that the actual presence of bile in the evacuations is not therefore a proof that the disease is not cholera. *Purging* has rarely been found absent, in some degree at least; but we are cautioned against placing too implicit reliance upon the statements of the patient on this point; however, it appears, that, after the first fœcal evacuation, the discharge is of a totally different nature, so that, on dissection, the intestines shall be found to contain no fœcal matter. The dejections vary much as to uneasiness and urgency; generally speaking, they are not attended with much pain or tenesmus; the quantity of watery matter is generally great, but not always so in those cases where the debility and exhaustion is most remarkable; and, as we before said, the return of fœcal evacuations is always favourable.

The *animal functions* do not participate so much in the disturbance of the system as might be anticipated. *Spasm* of the voluntary muscles, which has been looked upon as characteristic of the disease, is not unfrequently wanting, and they are chiefly found in those cases attended with violent commotion of the system, and are consequently more common among Europeans than among the natives, and rather in the robust than the weakly. In the most dangerous form of cholera it is in general wanting. The muscles of the extremities are those usually affected; the toes and calves of the legs first, then the fingers and arms, and lastly those of the trunk. Hiccough, when it occurs, is not at all indicative of danger. Retraction of the muscles of the abdomen is remarked as a frequent symptom: these contractions are not permanent, but are always attended with pain; twitchings of the muscles have occasionally been observed after death.

The *collapse of the circulation* is, of all the symptoms of cholera, that which is most invariably present, so much so, as to render it the distinctive character of the complaint; but the period at which this collapse takes place is various: on the accession of spasm or vomiting, if small and accelerated, it suddenly ceases to be distinguishable; yet, in this condition, the patient will sometimes live many hours; on the cessation of the spasm or vomiting, the pulse will sometimes return to the wrist; the superficial arteries and veins are not always collapsed even when the pulse has ceased: if these vessels are opened, the blood contained in them flows out; they then collapse, and no more can be taken away.

Of *Thirst* and *Heat in the Epigastrium* much need not be said; they are pretty constant symptoms; yet, though the thirst is great, there is no want of moisture in the mouth, and though there is burning heat within the mouth, the lips are cold and blanched. This symptom must indeed be distressing when we learn that even medical men, although aware that cold water is almost certain death, cannot refrain from seeking it. The *skin* is always, or nearly always, cold; it has occasionally been observed to become warm just before death, but then the warmth is confined to the trunk and head; and wherever it is *partial*, it is a fatal symptom. The sensation on touching the skin of a person in this disease resembles that communicated by a dead body;—and it is insensible to boiling water or mineral acids.

The *countenance*, in cholera, is very remarkable, and it appears shrunk in every case; but, of course, in different degrees of intensity,—it is, in fact, the countenance of death.

*Respiration* is not usually affected early in this disease; but it becomes slower as the complaint advances,—in one instance only occurring seven times in the minute; but it does sometimes happen that the muscles of respiration are affected with spasm, especially in Europeans, and then that function is as much disturbed as in a fit of asthma.

*Restlessness* is more observable with European than native patients; but moral causes may have some influence in this respect: it is always an alarming symptom when present, though its absence is not therefore to be considered as affording grounds for a favourable prognosis.

It does not appear that the *functions of the sensorium* are disturbed, or, at least, are very rarely so; coma has often been represented as a symptom of cholera, but this is inaccurate; the patient, indeed, feels great difficulty in overcoming that retirement within himself, which constitutes so remarkable a feature of the disease, but, if excited to speak, his answers are clear and precise, in this respect resembling what is often met with in cases of tetanus, hydrophobia, &c.

*Syncope* is not a common symptom in cholera, and, when met with, has usually occurred on the invasion of the disease. *Deafness* is sometimes the very first symptom that has developed itself.

When recovery from cholera takes place, it is generally very rapid, so much so, says Mr. Scot, as perhaps to be decisive of the disease being unconnected with any organic lesion. Among the natives, this is remarkably the case; but it is to be remembered that they are little liable to inflammatory affections: in Europeans, recovery is neither so sudden nor so perfect; but, on the contrary, the sequelæ are very various: among the most common, are affections of the brain, liver, intestines, or stomach. When the disease is of long continuance, however, few either of the natives or Europeans recover without considerable difficulty: a return of heat to the surface and a rising of the pulse indicate a probable recovery from this epidemic; such is more particularly the case where the attack has been violent, and the symptoms decisive; but when these are, what our author calls, *negative*, by which we presume he means a difference only in degree of violence, the evolution of heat and the return of arterial action are among the last of the functions restored. The secretion of urine, as well as all the natural secretions, being nearly suspended, in many instances the catheter was introduced under the idea that the absence of urine was owing to *suppression* (Mr. Scot evidently means *retention*, for no man would pass a catheter to remedy a *suppression* of urine); but, when the secretion of this fluid is not suspended, it is afforded in very small quantity, and is very limpid and clear. Some of these latter cases were observed to be as replete with danger as when the secretion was entirely suppressed; however, when that had been the case in the commencement of the attack, and the secretion became *restored*, recovery might be confidently expected. Instances are recorded where there had been no urine secreted for upwards of fifty hours.

The condition of the blood and the state of the circulation are singularly uniform in this disease: the Medical Board considered it so desirable to attain correct information upon these points, that circular letters were addressed to about thirty medical officers, whose experience was supposed to be the most extensive: their attention was directed especially to the following considerations;—1st, the influence which the state of the blood might be supposed to have in producing the symptoms; 2d, the color of the blood extracted from the vein of a person labouring under the disease; 3d, the color of the blood after a certain quantity had been obtained, and the effect which this alteration of color might appear to have on the patient; 4th, where arteriotomy had been practised, the color of the arterial blood; and, lastly, the period, from the first attack of the disease, at which blood had been taken away.

It is established by a vast mass of concurrent testimony that the blood, in cholera, is of a dark color and thick consistence; the change in color depending upon the time that the symptoms have existed. The abstraction of blood has also been universally found to be difficult and uncertain: when drawn, it is said to be very generally destitute of serum, is never buffy, but coagulates quickly. There are, however, exceptions to this last circumstance. It is observed, in a majority of the Reports, that, after some quantity has been withdrawn, the blood becomes lighter colored, and less thick, the circulation reviving at the same time. We need scarcely say this is very favourable, though such effects have not followed all successful bleedings: but we are informed that the blood is found less changed in appearance in those cases which commence with symptoms of excitement, than where a collapsed state of the system has been observed at an early period. On dissection, the blood has occasionally been found as dark on the *left* as on the *right* side of the heart; in the temporal artery, too, when opened, the blood has been observed to be dark and thick; but it has not been possible, in general, to obtain much blood by arteriotomy: nay, the brachial artery has been opened, without any blood flowing. When re-action has become established, the buffy coat has been sometimes seen. Mr. Scot laments that the state of the respiration, as connected with the condition of the blood, has not been more accurately ascertained, since the coldness of the body would lead to the conclusion that the temperature of the blood is lowered in cholera; nor can the thickness of the blood be always explained by the quantity of watery discharges from the intestines and surface of the body, since, after death, the bowels have been found filled with fluid, though no vomiting or purging has occurred; and yet in these cases the blood drawn has been equally thick.

With respect to the termination of cholera, it may be said that the fatal result is never averted by the mere efforts of nature; nay, the delay of a few hours places the patient beyond the reach of art. Indeed, our author informs us that some medical men doubt the power of medicine altogether in the cure of this disease; but, from its never-failing mortality among the native population, we are inclined to believe that such an opinion is at least exaggerated, and an inspection of the annexed Returns will, we think, sufficiently bear us out in the conclusion that the science of medicine is not applied in vain to the relief of this most formidable scourge: for example, Mr. Searle, of Marantoddy, says, that of twenty-eight villagers attacked with cholera, twenty-six died, the other two recovered by his assistance. The Ameen of Ganjam writes thus—"The people who get the cholera morbus never recover; to them death is certain." The family of a wealthy pair at Travancore, consisting of nineteen persons, were all cut off, save one, in a few hours. In the great majority of cases, a general suspension of the natural, and a gradual cessation of the vital, functions, is the ordinary termination of cholera: sometimes death ensues from supervening inflammation of some of the viscera,—either hepatitis, enteritis, or dysentery.

Mr. Scot next proceeds to consider the diagnosis of cholera. The reader who has attentively perused the preceding pages, will not, we conceive, find much difficulty attending this task. The complaint with which it is most apt to be confounded, is the bilious cholera; but the coldness of the surface, the collapsed features, the sunken state of the circulation, the different nature of the evacuations, and the depression of spirits, will form such a clear line of distinction, as to render mistake very unlikely; and it only requires attentive discrimination, when the disease is epidemic, to distinguish other spasmodic or nervous attacks, or the accession of the cold stage of common fever, from it.

Of the principal appearances on dissection, the following is a sketch; though we must premise, that these examinations are made under some disadvantage in a hot climate, and, from the prejudices of the natives, have only taken place to a very limited extent among them. The observations, therefore, principally apply to the bodies of European soldiers. The external appearance of the body is livid and shrunk; there is no particular tendency to putrefaction produced by the disease, nor are there any particular morbid appearances to be detected in the cavities lined by the serous membranes, or in those membranes themselves. It is in the surfaces lined by *mucous membranes*, that the signs of disease are to be found. In the lungs, the chief morbid appearances are, that they seem either gorged with blood or collapsed into a very small bulk, leaving the cavity of the thorax nearly empty. In the heart, the only remarkable appearance has already been mentioned,—namely, that the left, as well as the right, cavities have been found distended with dark-colored blood. The external appearance of the viscera of the abdomen has nothing peculiar about it: occasionally the surface of the intestines is more vascular than usual; at other times they appear blanched. The intestinal tube is most commonly found distended with air, and containing whitish, or sometimes greenish, turbid fluid; no fœcal matter is found in the canal; the duodenum and jejunum have been found filled with an adherent mucus, sometimes entirely free from it, at others perfectly healthy. It is very rare to find any traces of bile in the intestines. Sanguineous congestions are more common in the bowels than in the stomach. About the condition of the liver nothing very decisive is said; but the gall-bladder is always found to contain bile, and is often quite distended with it. The urinary bladder is invariably empty and contracted.

The spleen offers nothing particular; the vessels of the mesentery are gorged with blood. In the brain, congestion is sometimes said to have been observed; but this does not seem to be clearly made out. The spinal canal has only been examined in one case, and there its sheath was highly inflamed: that case was said, however, to have been a mixed one.

From the above enumeration we must concur in opinion with our author, that, in a pathological point of view, the information derived from dissection is of a negative nature only. We beg leave to omit Mr. Scot's explanation of the pathology of this disease, because our space, we conceive, is better occupied with the detail of facts connected with it; and from these facts our readers will be enabled to form to themselves as good a theory as any that appears to have been yet offered upon this subject.

We proceed, therefore, to the predisposing causes. Debility, either constitutional or consequent upon previous disease, seems to stand first upon this list: patients under the full influence of mercury, and also pregnant women, have been seized with it: in short, all causes which conduce to nervous or cachectic diseases, predispose to cholera; and, consequently, the poorer classes suffer the most. Of remote causes, we have nothing positive to affirm; for, surely, errors of diet, vicissitudes of weather, fatigue, exposure, and the depressing passions, are not peculiarly the remote causes of this, but apply to every disease; and, in many epidemic visitations of cholera, in numberless instances most of these remote causes have appeared to be innocuous. As an example, our author observes, that the labourers at the public works at Madras, on one occasion, who were well fed, clothed, and lodged, and did not work particularly hard, suffered most severely from it; whilst a body of men, employed in clearing out the beds of stagnant and very putrid waters, and who were exposed to heat and rain, entirely escaped. Again, of two corps of an army, placed precisely under the same circumstances, one has escaped, the other suffered greatly. At one time, a regiment marches through a district labouring under cholera, and does not experience the disease; at another, the corps suffers severely, though the disease does not exist among the inhabitants. Neither situation nor atmospheric changes would seem to have any influence upon the production of the disease, though a corps in the field or on the march is certainly more liable to the disease than when in quarters. There is no instance on record of a case occurring on-board ship, prior to communication with the shore; but it has often appeared on-board ships that have sailed from India. Finally, it has sometimes supervened on the use of neutral purging salts, or from drinking unwholesome liquors. From all which considerations, and many others, says our author, the immediate cause of Cholera, or the epidemic influence, as it is called, will naturally be sought either in the atmosphere, in the soil or its productions, or in a power *sui generis*, capable of propagating itself, and therefore involving the question of its contagious nature.

Mr. Scot proceeds to examine each of these causes at some length; but in no instance is the testimony so conclusive as to merit particular mention. We think also, that our author has bestowed more time and space than was necessary in considering the effects of solar influence, and of electricity, in giving origin to cholera. These topics have, perhaps, in India, acquired a reputation founded upon the high opinion entertained of Mr. Orton's publication; but we, who view the matter from a distance, cannot, upon looking carefully over the evidence offered in support of this theory, allow any weight to it. With respect to the influence of deleterious food, it may be necessary to say, that Dr. Tytler has ascribed the complaint entirely to the use of what has been called "ooze rice;" but our author gives to us conclusive reasons against this explanation of the origin of cholera.

Lastly, we come to the consideration of the contagious nature of the disease; a question always difficult, and which we find here involved in at least its usual quantity of perplexity. Mr. Scot first defines the sense in which he understands the words contagion and infection, restricting the import of the first to actual contact with the deceased, and applying the term infection to the communication of a disease from one person to another, through the medium of the atmosphere. Our author, however, does not rigidly adhere to this definition, but subsequently seems to employ these terms indifferently. We find that the great majority of medical men in India deny that cholera is either infectious or contagious; but the statements given by our author are of the most contradictory description. One thing is certain, that its march from north to south was uniformly progressive; but, when we come to *particular* evidence as to any of its local visitations, we begin to be perplexed: thus it has frequently happened that it has manifested itself at a place before uninfected, in the attack of a person who came from a place where it existed; and the instances of its appearing immediately after the arrival of troops suffering from it, at situations where it did not before exist, are very numerous: not less than ten or twelve strongly-marked illustrations of this fact are mentioned. Cholera likewise has been traced along a whole street, and through whole families; it has been observed to travel along the main road, affecting the villages on each side of it, but leaving those at a distance untouched.

The evidence in favour of the contagious nature of the disease, as it respects the intercourse between individuals, is also strong in many respect. The cases of servants attending their masters, of wives, their husbands, &c. are very frequently met with; and it appears that thirteen medical officers on the Madras establishment died of the disease, and between fifteen and twenty, though affected with it, recovered: in every instance, these officers had been engaged with patients labouring under cholera. The reasons for doubting the contagious nature of the disease, are chiefly derived from theoretical views; but the escape, in so many instances, of the attendants of the sick under severe visitation of the disease,—the fact of the total immunity of the surgeons, who in many instances slept in their hospitals, must be considered as very strong evidence. The following short extract is, perhaps, among the strongest proofs adduced on this side of the question:—

"Mr. Acting-surgeon Gibson, in reporting on a late attack, (April 1823,) experienced by the 69th regiment, at Wallajahbad, observes,—'I had ninety-two admissions, and increased the establishment of servants to double; I lived in the hospital, amidst the sick, day and night; and yet neither I myself, nor any of the servants, got the disease: but the hospital serjeant's wife, living in a retired room, not near any disease, had a severe attack. The disease came on suddenly with a hot land-wind, and went off as suddenly when it ceased. At my suggestion, that wing of the regiment in which the disease prevailed the most, was encamped on a piece of high ground in the neighbourhood, and not a case came in from the camp, although every intercourse imaginable was kept up between it and the barracks. No steps were taken to prevent contagion when this wing of the regiment returned to the same barracks, and yet no cholera has since been heard of.'"

We shall offer no comments on the above evidence; it is probable that both accounts may be reconcileable to truth: the different degrees of intensity in which an epidemic disease prevails, may superadd a contagious character to it in one instance, and not in another. Such appears to us at least probable, and borne out by something like analogy in typhus, and other diseases of that character; but we do not mean to insist upon this explanation.

We pass by a long discussion upon the proximate cause of cholera, as totally unprofitable, and proceed to consider the *prognosis*, which, in this formidable disease, is not only too often fatal, but is still more frequently very uncertain. As a general remark, it may be said to depend a good deal upon the type of the disease: if the symptoms are *apparently* violent, if the pain and irritation be great, and the spasms considerable,—if there is much excitement of the system, and the case is seen early, a favourable event may be expected, and medicine can do much; but the state of collapse, with coldness of the surface, where there is little pain, little vomiting or purging, and a trifling degree of spasm, indicate a state of imminent danger; and here minutes must be looked upon as hours, and hours as days, with reference to other diseases. The most favourable of all signs is the restoration of the pulse, and the gradual and general return of heat; but, even under these last circumstances, we are never sure that, from some sudden or hidden movement of the system, or from some imprudence of the patient, the pulse may not all at once sink, and death ensue. Thus we see, in truth, the prognosis is very generally fatal, and almost always uncertain.

[To be continued.]

